# Prolactin Monitoring and Management Re-Audit in an Acute Mixed Adult Inpatient Psychiatric Unit

**DOI:** 10.1192/bjo.2026.11743

**Published:** 2026-06-30

**Authors:** Syeda Maryam Bilgrami, Anousheh Bilal, Rafi Mahmoud, Ibrahim Habash

**Affiliations:** Berkshire NHS Foundation Trust, Reading, United Kingdom

## Abstract

**Aims:**

Antipsychotic medications acting on dopamine D2 receptors are commonly associated with hyperprolactinaemia, which can lead to distressing symptoms such as galactorrhoea, menstrual irregularities and sexual dysfunction, as well as longer-term complications including osteoporosis.

This re-audit aimed to assess adherence to Trust guidance on baseline prolactin monitoring and management of elevated prolactin level.

Trust guideline on antipsychotic induced hyperprolactinemia states the following: “Pre-treatment screening is vital in helping to determine whether or not a subsequent elevated prolactin level is due to medication”.

**Methods:**

A retrospective audit was conducted for all patients admitted to Rose Ward, Prospect Park Hospital, between 1 June and 31 August 2025.

Audit standards were: (1) all admitted patients should have serum prolactin measured on admission; and (2) prolactin levels >1000 mIU/L should prompt clinical intervention. Data were obtained from ICE blood test records, with RiO used to identify documentation of blood tests, refusals, and management of raised prolactin levels.

**Results:**

Of 42 patients admitted during the audit period, 76% (n=32) had serum prolactin measured on admission, demonstrating improvement compared to 69% in the 2024 audit. Of the remaining patients, six refused blood tests and four had no documentation of prolactin measurement.

As per Standard 2, four patients had prolactin levels >1000 mIU/L,100% had clinical intervention, action was taken as follows:

Patient A was a known case of prolactinoma and already under endocrinology

Patient B & C were already on antipsychotics on admission (Paliperidone and Risperidone). They were monitored and reported no symptoms. Macroprolactin was pending but not followed up for Patient B.

Patient D was already known to have hyperprolactinaemia as she was on zuclopenthixol and aripiprazole 5mg. The plan was to continue with 5mg and monitor her prolactin levels.

**Conclusion:**

This re-audit demonstrates improved compliance with baseline prolactin monitoring and appropriate identification and management of hyperprolactinaemia. Nevertheless, poor documentation of clinical symptoms highlights the need for improved clinician awareness and structured assessment.

Action plan: Enhance junior doctor education with Prolactin Monitoring guidance added to FY1 Doctor Induction Handover Booklet + Presentation for incoming rotation (Dec 2025). Posters on prolactin monitoring and management distributed to the On-Call room, Doctor’s mess and Rose Ward clinic room, and the introduction of a symptom screening tool adapted from the Glasgow Antipsychotic Side-Effect Scale to support holistic assessment and management.

